# “Oh, My God! My Season Is Over!” COVID-19 and Regulation of the Psychological Response in Spanish High-Performance Athletes

**DOI:** 10.3389/fpsyg.2021.622529

**Published:** 2021-03-24

**Authors:** Juan González-Hernández, Clara López-Mora, Arif Yüce, Abel Nogueira-López, Maria Isabel Tovar-Gálvez

**Affiliations:** ^1^Health Psychology/Behavioural Medicine, Research Group (CTS−0267), University of Granada, Granada, Spain; ^2^Human Development and Family Science, University of Missouri-Columbia, Columbia, MO, United States; ^3^Department of Sports Management, Faculty of Sports Science, Eskisehir Technical University, Eskisehir, Turkey; ^4^Department of Health and Sport, European University of the Atlantic, Santander, Spain; ^5^Department of Health and Sport, International Ibero-American University, Campeche, Mexico; ^6^Department of Health and Sport, International University of Cuanza, Cuito, Angola; ^7^Biomedical Group (BIO277), Department of Nursing, Faculty of Health Sciences, University of Granada, Granada, Spain

**Keywords:** distress tolerance, anxiety, depression, athletes, COVID-19

## Abstract

**Background:** In an unprecedented situation of interruption of the sporting dynamics, the world of sport is going through a series of adaptations necessary to continue functioning despite coronavirus disease 2019 (COVID-19). More than ever, athletes are facing a different challenge, a source of discomfort and uncertainty, and one that absolutely alters not only sports calendars, but also trajectories, progressions, and approaches to sports life. Therefore, it is necessary to identify the levels of psychological vulnerability that may have been generated in the athletes, because of the coexistence with dysfunctional responses during the COVID-19 experience, and which directly influence the decrease of their mental health.

**Methods:** With a descriptive and transversal design, the study aims to identify the state of the dysfunctional psychological response of a sample of Spanish athletes (*N* = 284). The DASS-21 (Depression, Anxiety, and Stress Scale), Toronto-20 (alexithymia), and Distress Tolerance Scale questionnaires were administered to a sample of high-level Spanish athletes in Olympic programs.

**Results:** The results suggest that the analyzed athletes indicate high levels of dysfunctional response (e.g., anxiety, stress, depression, and alexithymia) when their tolerance is low. In addition, the variables show less relational strength, when the capacity of tolerance to distress is worse and age is lower. At the same time, the greater the anxiety and uncertainty are, leading to more catastrophic and negative thoughts, the younger the athletes are.

**Conclusions:** It is clear that both age and tolerance to distress are considered adequate protective factors for psychological vulnerability in general and for associated dysfunctional responses in particular. Moreover, the psychological resources offered by more experienced athletes are also a guarantee of protection against negativity and catastrophism.

## Introduction

Emotional stability in athletes is extremely necessary. They need to focus their efforts on clear, concrete, and planned objectives. Temporarily, athletes find in competitions possibilities to measure themselves and their opponents. The lack of all these details in an athlete's life, even with their constant orientation toward sporting challenges, increases psychological processes that lead to uncertainties, directly influencing their personal health and professional dedication. In the situation of the global pandemic by the coronavirus disease 2019 (COVID-19), the psychological resources of the athletes have been questioned; added and multiplied pressure is created that involves experiencing it in a traumatic way in the present and with possible repercussions in the future. Professional sports have been one of the most punished contexts, where all the possibilities of competing have disappeared (UEFA Soccer CUP, World Championships in all modalities, Tour of France and Giro of Italy, or JJOO have been canceled).

Generally, the experiences of high-performance athletes (e.g., sporting demands, constant challenges) require a great emotional and psychosocial stability, which strengthens them in constant competitive experiences (e.g., managing successes, resilience, accepting failures) (Cece et al., 2019), but the unexpected coexistence with an unknown disease without treatment, the inability to determine the psychosocial and economic repercussions, and the uncertainty of how to deal with the virus are all excessively traumatic situations, which turned them into a very high-risk population that will require a functional response and ready and in the best possible conditions for the future (Costa et al., 2020).

Préville et al. (1995) described the psychological distress syndrome as a combination of five factors (depression, anxiety, anger, cognitive problems, and somatization), understood as second-order factors, which also reflect the non-specific coexistence of differential distress symptoms in all these factors. Other researchers have defined it as a process of breakdown (Frank, 1973) or psychological suffering (Vélez et al., 2013; Den Hartogh, 2017), describing both somatic complaints and psychological symptoms. Furthermore, the most recent reports have demonstrated the relevance of latent but ambiguous psychophysiological symptoms, reflecting possible links with the presence of physiological and emotional disturbances (Haeberlein et al., 2020), including alexithymia as a major health risk factor (Davydov, 2017).

**Figure 1:**
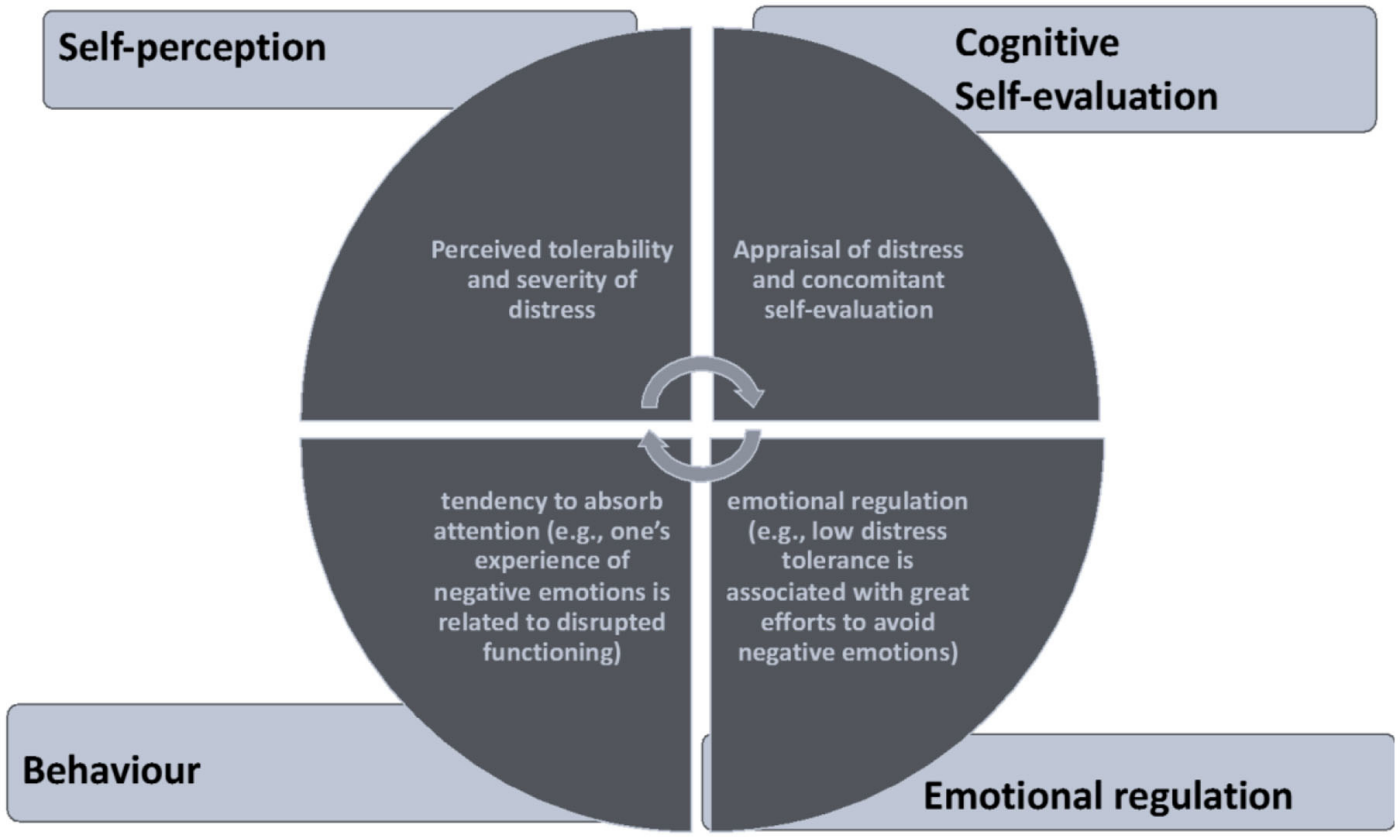
Distress tolerance model (Simons and Gaher, 2005).

Distress tolerance is considered to be the perceived ability to endure negative emotional and distressing internal states caused by some type of stressor (Simons and Gaher, 2005; Bardeen and Fergus, 2016) (see Figure 1). It influences and is altered by a variety of psychological processes involved in cognition (e.g., attention distortions, rumination), emotional responses (e.g., emotional lability) (Honkalampi et al., 2018), physical responses (e.g., digestive distress, sleep disturbances), or social responses (e.g., perceived social support, isolation) (Drapeau et al., 2012). Thus, people with low distress tolerance perceive distress as unbearable, unacceptable, and uncontrollable, and to be overly reactive to stress and distress (Sandín et al., 2017).

This study is important for investigating individual differences that impacts an athlete's well-being. This makes necessary to identify the levels of psychological vulnerability in athletes that may be generated by living together with dysfunctional responses during and after the COVID-19 experience, directly associated with the decrease in mental health. In the same way that Mannes et al. (2019) mention how athletes feel and suffer intense unrest when the inevitable sporting withdrawal (e.g., due to age, injury) occurs, in this “forced withdrawal” the response is much more unspecific and contradictory.

Issues such as the intensity, frequency, and duration of these processes will influence athletes to develop a lower tolerance to distress (Zvolensky et al., 2010), protecting their defensive resources to cope effectively with anxiety, stress, or depression (Cheung and Yip, 2015) if this tolerance is high (Boffa et al., 2018). Sporting experience is another important issue, as high-performance athletes are used to performing under pressure, and in the face of the paralysis and uncertainty generated by the pandemic, many of their psychological strengths have been put to the test.

Both situational factors and individual differences exert their influence on the perception of negative stress (Turner et al., 2019a) or in the absence of words to express one's emotions and difficulty recognizing one's own emotions and feelings along with the inability to express them to others (e.g., alexithymia) (Eccles et al., 2011), highlighting studies that associate it with the motivational orientation of mastery transmitted by coaches and peers, in the face of perceived lack of capacity (Pensgaard and Roberts, 2000), high sport pressure (Roberts and Woodman, 2017), or vital risk (Woodman et al., 2008). Similarly, cognitive–behavioral (e.g., negative thoughts, irrational beliefs) arguments have served to describe the distress response to contextual influence through the mediation of cognitive response (Nixdorf et al., 2013; Turner et al., 2019b).

Studies that have sought to explain the connections between depressive responses in sports populations have described major events in sports life (e.g., injuries, failures) (Frank et al., 2015; Putukian, 2016), self-esteem (Armstrong and Oomen-Early, 2009), and, of course, anxiety (Gorczynski et al., 2017). In addition, it has been reported that athletes show lower indicators of depression than non-athletes (Brand et al., 2013), which appears more in female athletes (Wolanin et al., 2016) and young athletes (Junge and Feddermann-Demont, 2016). Similarly, while anxiety is one of the most studied variables in the psychological interpretation of the athlete, it has been associated with perceptions of distress through the influence of perfectionist beliefs (Madigan et al., 2017), rumination (Grossbard et al., 2009), or self-regulation (Steiner et al., 2010).

A significant connection between depressive symptomatology and distress has been understood (Reardon et al., 2019), as well as negative coping (Madigan et al., 2018), rumination (Walton et al., 2020), or catastrophism (Rice et al., 2016) in “regular” sport situations. Under the COVID-19 pandemics, this relationship is more evident. Undoubtedly, the pandemic has changed many athletes' behaviors. Recent studies have provided evidence of this relationship. See, for example, the appearance of gambling problems (Håkansson et al., 2020), social distancing, and loneliness (Graupensperger et al., 2020; Senişik et al., 2020) or adaptations in mental health (Yousfi et al., 2020).

Therefore, the aims of this study are (1) to describe how athletes are coping with the situation in COVID-19, regarding their cognitive and emotional response; we expect to find significant levels of indicators of dysfunctional psychological response (anxiety, stress, depression, alexithymia, and distress); and (2) to study participants' distress tolerance and its relationship with emotional vulnerability (high anxiety, stress, and depression). We expect that athletes showing higher distress tolerance will report lower scores in stress, alexithymia, anxiety, and depression.

## Methods

### Sample and Procedure

We chose a transversal and non-randomized design to assess anxiety, stress, and depression (vulnerability factors in psychological health) in a sample of Spanish athletes (*N* = 284). The average age was 24.26 (SD = 6.83) years, of which 78.3% were men and 21.7% were women. The range of sport experience was from 9 to 22 years [mean = 15.02 (SD = 4.86) years]. Different professionals (belonging to professionals' leagues), Olympians (competing in the summer and winter Olympic Games), and athletes from other sports (Table 1) were represented. From March to June 2020, athletes were contacted both at their workplaces and in person to conduct this research. Participants completed an online Google forms questionnaire we developed. All participants completed a consent form approved by the Ethics Committee of the University of Granada (ID: 1494/2020). Participants were required to read, accept, and sign it voluntarily.

**Table 1:** Descriptive data on statistical variables.

***N* = 284**	**Range**	**%**
Age (years)	18–31	
**Gender**		
Male	222	78.3
Female	62	21.7
Sport experience (years)	9–22	
**Sport modalities**		
Olympic sports (athletism, swimming, combat sports, cyclist,…)	96	33.8
Professional sports (soccer, basketball, handball, tennis,…)	134	47.1
Other sports (running, billiard, chess,…)	54	20.1
**Competition level**		
Under 23	159	56.0
Senior	125	44.0
	**Range**	**Mean (SD)**
Anxiety	0–21	15.73 (3.61)
Depression	0–21	13.15 (3.11)
Stress	0–21	16.38 (3.04)
Alexithymia	20–100	49.37 (12.13)
Distress	15–75	54.02 (4.62)

### Measures

#### Dysfunctional Psychological Response

The Spanish version of the questionnaire Depression, Anxiety and Stress Scale (DASS-21) (Daza et al., 2002) was used to measure emotional distress in three subcategories: stress, anxiety, and depression. Self-report with 21 items (seven for each category) was based on a score of four points: (0) “not applied to me at all” to (3) “applied to me a lot.” The higher the score, the more severe the indicator is. Global score demonstrated a good internal consistency (α = 0.87), as well as good depression (α = 0.84), anxiety (α = 0.86), and stress (α = 0.80) scores.

#### Tolerance to Distress

Stress Tolerance Scale was administered in its Spanish version (ETD; Sandín et al., 2017). It is a 15-item self-report designed to assess the degree to which individuals experience and cope with psychological distress, on a 5-point scale [(1) “strongly agree” to (5) “strongly disagree”]. Consistency was high (α = 0.86). The higher the score was, the more resources participants showed for managing distress responses.

#### Alexithymia

We used the Toronto Scale of Alexithymia in its Spanish adaptation (TAS-20; Páez et al., 1999) to measure inability to control and recognize emotions. The 20 items in the questionnaire are scored using a Likert scale from (1) “strongly disagree” to (5) “strongly agree,” The score obtained is considered alexithymic if the person obtains a score equal to or greater than 61. We obtained an adequate consistency level (α = 0.84).

### Data Analysis

We used the IBM SPSS Statistics 25 software to run the statistical analyses. We calculated descriptive measures (tendency and Kolmogorov–Smirnov) of stress, anxiety, depression, distress, and alexithymia. We also calculated the internal consistency (Cronbach α and Cohen *d*) of stress, anxiety, depression, distress, and alexithymia. We performed *t* tests to study the mean differences of stress, anxiety, depression, distress, and alexithymia between professionals, Olympians and other sports athletes. Showing the linearity relationships between stress, anxiety, depression, distress, and alexithymia, we calculated bidirectional correlations (Pearson). Finally, a multiple regression analysis was performed. Distress tolerance was the dependent variable, and stress, anxiety, depression, and alexithymia were the predictors (5.000 bootstrap resamples to establish the significance < 0.05).

## Results

The descriptive data indicate that the scores on dysfunctional psychological response of the selected sample were above the normal average scores for each variable (Table 1). Only alexithymia and depression showed levels around the mean.

Relationships of the dysfunctional response were analyzed through the calculation of the partial correlations among distress and all other variables, controlling the effect of the remaining ones. In addition to the “zero order” correlation and the partial correlation of each of the predictor variables with distress, the semipartial correlation was calculated (Table 2). All the correlations were positive and significant. Correlations between distress and anxiety and between distress and stress were the highest.

**Table 2:** Partial correlations between distress and anxiety, stress, depression, and alexithymia, controlling the effects of variables (*n* = 284).

	**Anxiety**		**Depression**		**Stress**		**Alexithymia**	
	**Partial**	**Semipartial**	**Partial**	**Semipartial**	**Partial**	**Semipartial**	**Partial**	**Semipartial**
Distress tolerance	0.436^**^	0.378^**^	0.245^**^	0.203^**^	0.346^**^	0.278^**^	0.304^**^	0.285^**^

Linear and differential relationships (Table 3) between the variables studied showed that as the participants increase in age, depression, anxiety, alexithymia, and stress scores decreased significantly, both when the tolerance to distress was low and high. Likewise, Distress Tolerance showed positive and significant relationships with depression, anxiety, alexithymia, and stress, being stronger when tolerance distress was low. In addition, significant differences in the levels of anxiety, depression, stress, and alexithymia were shown, mainly when tolerance to distress was low.

**Table 3:** Differential and linear relations, according distress tolerance levels.

***N* = 284**	**Range**	***d***	**Low distress tolerance**						**High distress tolerance**					
			**Mean (SD)**	**1**	**2**	**3**	**4**	**5**	**Mean (SD)**	**1**	**2**	**3**	**4**	**5**
Sport experience	9–22	0.75	7.41 (7.25)	–					16.78 (6.04)	–	−0.71^**^	−0.59^**^	−0.76^**^	−0.59^*^
Anxiety	0–21	0.79	14.03 (2.96)	−0.74^**^	(0.85)^a^				11.42 (2.75)^c^		(0.86)^a^	0.43^**^	0.47^*^	0.49
Depression	0–21	0.83	16.24 (3.71)	−0.54^*^	0.76^**^	(0.82)^a^			10.07 (3.38)^b^			(0.81)^a^	0.39^*^	0.31^*^
Stress	0–21	0.85	17.06 (2.95)	−0.61^**^	0.74^**^	0.76^**^	(0.83)^a^		13.70 (2.80)^b^				(0.84)^a^	0.34^*^
Alexithymia	20–100	0.86	59.91 (14.51)	−0.48^*^	0.68^*^	0.74^**^	0.58^**^	(0.88)^a^	38.84 (7.31)^c^					(0.86)^a^

* p < 0.05;

** p < 0.01;

a Cronbach α; means differences (Student t);

b p < 0.01;

c *p < 0.05; d: Cohen reliabilty*.

Predictive analysis (Table 4) revealed different significant relationships about dysfunctional response, depending on whether tolerance to distress was high or low [depression (*F*_(4, 279)_ = 67.34, *p* < 0.01), anxiety (*F*_(4, 279)_ = 70.16, *p* < 0.01), stress (*F*_(4, 279)_ = 68.03, *p* < 0.00)] or low [depression (*F*_(4, 279)_ = 69.68, *p* < 0.01), anxiety (*F*_(4, 279)_ = 71.56, *p* < 0.00), stress (*F*_(4, 279)_ = 68.36), and alexithymia (*F*_(4, 279)_ = 67.12, *p* < 0.01)]. More specifically, predictive relationships were stronger for dysfunctional response when tolerance to distress was lower.

**Table 4:** Regression analysis over anxiety, depression stress, and alexithymia, according distress tolerance levels.

**Low distress tolerance**				**High distress tolerance**			
**VD: DEPRESSION**							
***R***^**2**^ **= 63.7% (*****p*** **< 0.00)**	***β***	***t***	***p***	***R***^**2**^ **= 61.2% (*****p*** **< 0.00)**	***β***	***t***	***p***
(Constant)		0.54	0.48	(Constant)		0.52	0.42
Sport experience	−0.66	−3.01	0.02^*^	Sport experience	– 0.57	−2.82	0.26
Alexithymia	0.61	2.67	0.02^*^	Alexithymia	0.39	2.27	0.26
Anxiety	0.75	6.92	0.00^**^	Anxiety	0.68	6.41	0.00^**^
Stress	0.58	4.82	0.00^**^	Stress	0.46	4.71	0.00^**^
**VD: ANXIETY**							
***R***^**2**^ **= 63.1% (*****p*** **< 0.00)**	***β***	***t***	***p***	***R***^**2**^ **= 56.68% (*****p*** **< 0.00)**	***β***	***t***	***p***
(Constant)		−2.08	0.21	(Constant)		−2.34	0.19
Sport experience	−0.12	−2.50	0.03^**^	Sport experience	−0.39	−2.52	0.21
Alexithymia	0.43	2.14	0.01^*^	Alexithymia	0.27	2.05	0.21
Depression	0.49	4.98	0.00^**^	Depression	0.21	4.37	0.02^*^
Stress	0.47	5.36	0.00^**^	Stress	0.32	5.86	0.01^*^
**VD: STRESS**							
***R***^**2**^ **= 58.3% (*****p*** **< 0.00)**	***β***	***t***	***p***	***R***^**2**^ **= 55.6% (*****p*** **< 0.00)**	***β***	***t***	***p***
(Constant)		−3.82	0.27	(Constant)		−3.49	0.24
Sport experience	−0.26	−3.73	0.00^**^	Sport experience	−0.56	−4.01	0.31
Alexithymia	0.24	−2.76	0.02^*^	Alexithymia	0.49	1.23	0.56
Depression	0.37	4.93	0.00^**^	Depression	0.66	5.83	0.02^*^
Anxiety	0.53	6.26	0.00^**^	Anxiety	0.64	5.35	0.01^*^
**VD: ALEXITHYMIA**							
***R***^**2**^ **= 58.3% (*****p*** **< 0.00)**	***β***	***t***	***p***	***R***^**2**^ **= 62.7% (*****p*** **< 0.00)**	***β***	***t***	***p***
(Constant)		−03.64	0.34	(Constant)		−2.43	0.24
Sport experience	−0.17	−2.76	0.00^**^	Sport experience	−0.62	−1.62	0.12
Anxiety	0.37	2.61	0.02^*^	Anxiety	0.65	1.44	0.03^*^
Depression	0.61	3.47	0.00^**^	Depression	0.48	4.37	0.01^*^
Stress	0.52	6.15	0.00^**^	Stress	0.69	6.42	0.02^*^

* p < 0.05;

** *p < 0.01. VD, dependent variable*.

## Discussion

Results suggest that the athletes analyzed indicate lower levels of dysfunctional response (e.g., anxiety, stress, alexithymia, and depression) when their distress tolerance is higher. The more resources athletes show to withstand the distress, the weaker the appearance of anxiety, depression, and alexithymia is.

The first aim was to describe how athletes have perceived their cognitive and emotional response to the situation created by the COVID-19 pandemic. When facing a stressor, individuals experience an alteration of their psychophysiological activation level. This effect happens before the person is aware of the impact that the stressful situation has had on them (Chalmers et al., 2014). As in many other professional areas, being exposed to stressful sources for a prolonged and uncertain period of time triggers dysfunctional responses in sportsmen and sportswomen, which may involve an important risk of suffering psychological alterations or disorders (di Fronso et al., 2020).

Competitions cancelation, moving away from going to the locker room, and frequent training stoppage due to team mates or coaches positives in COVID are common issues sportsmen and sportwomen face right now (Arnold et al., 2018). Athletes experience uncertainty not only about competition and their sport career, but also about their personal lives (Schinke et al., 2020).

Another purpose of the present study was to establish relationships on how the tolerance distress is a psychological resource that protects from emotional vulnerability (high anxiety, alexithymia, stress, and depression). As stated in the hypothesis, athletes who report higher indicators of distress tolerance report a lower score for a dysfunctional psychological response such as stress, alexithymia, anxiety, and depression (Simons and Gaher, 2005).

Because of the constant presence of stressful events in sports, sportsmen should be used to deal with them and the positive and negative emotions that are frequently attached to sport competitions and practices (Laborde et al., 2016). However, this may not be the case. In fact, according to our data, an unusual situation such as the one we are living with, the COVID pandemic, generates a significant source of stress that accumulates to the regular level of stress sport performance involves. Athletes who do not have an adequate capacity to adapt and to cope with the high stress level may feel an increase in their stress level perception (Sukys et al., 2019).

Increased acceptance, risk-taking, self-regulation, and positive coping become possible transdiagnostic markers of psychological distress (Rice et al., 2020). Hence the important relevance of building on their sporting abilities, psychological resources that promote self-regulation, resilient resources, and adequate emotional coping that allow the appearance of a high tolerance to distress (Donohue et al., 2018).

However, our study has some limitations. Results may be influenced by the sample chose. Their competitive level (e.g., used to traveling, competing), the cultural heterogeneity of each sport (e.g., indoor sports, open spaces), or other personal circumstances of the athletes (e.g., being away from family or their usual training places) may have influenced the results. Even taking into account the difficulties of data collection (e.g., confined athletes and researchers), the size of the sample can be discussed, making it difficult to generalize the results obtained to other groups of athletes (e.g., team sports, individual sports,…). Therefore, we expect to replicate the study with more homogeneous samples (e.g., gender, age) and other performance levels (e.g., team competition level). Counting with the study transversal nature, it only allows the adjustment of the model in a certain period of time, which, although opportune, circumscribes the understanding of the causal process of the results to very similar situations. However, these results allow us to suggest future studies, both transversal studies that allow us to contrast the data obtained from similar samples (e.g., other countries, other types of athletes, by gender) and longitudinal studies that allow us to advance in the knowledge of the repercussions that the COVID-19 situation is generating in populations of athletes.

In addition, it is proposed to add cognitive and emotional variables (e.g., resilience, coping, motivation,…) to complete these results in the next research proposals. In this way, it will be possible to analyze the cognitive, emotional, and temperamental connections and their links, through new explanatory proposals or models that will make it possible to describe more precisely the functional and adaptive response of athletes to critical situations that affect their sporting life (e.g., sporting withdrawal, long-term injuries).

## Conclusions

The effective implementation of psychological health interventions, before and during crisis situations, is a unique opportunity for the functional to assess vulnerability versus empowerment. The supervision of studies in this line will provide medium- and long-term evidence of the response and psychosocial health in athletes, essential for the proper development of preventive measures and the anticipation of psychiatric and psychological care, as well as with the promotion and training of personal skills (e.g., specific training, mental training) to manage and preserve it.

Failure is an experience we may be facing every day. And it is not alien to anyone; we all have failures. The important thing here is to have sufficient reason to get up and either continue our efforts toward achieving the objectives, or reformulate them, or postpone them, or offer them a new time frame. The idea of failure is very much related to the figure of a loser. However, athletes who faced more failures used to be better prepared mentally, physically and technically to face their sport competitions. Fear of failure is one of the worst feelings that an athlete can experience, as when faced with the magnification of the situation that has occurred, the emotional reaction (e.g., shame, fear of social criticism, guilt) paralyzes him, leading him to respond in an altered, dysfunctional way that, in most cases, leads to a high suffering.

Teaching athletes to analyze the function of their negative emotions will bring them kindness (being kind, supportive, understanding themselves in times of pain in the face of self-judgment), feeling “human” (recognizing that failing and being imperfect is part of the human condition in the face of isolation) and empathic self-awareness (a balanced awareness of negative thoughts and emotions in the face of over identification).

Self-pity has the capacity to make us anticipate positive feelings in a more stable way than self-esteem (Leary et al., 2007; Neff and Vonk, 2009). The ability to forgive ourselves and be empathetic to ourselves (Sherman, 2014) helps us to stop comparing ourselves so much with others and to reduce our inner rumination or anger.

Despite the paucity of studies on samples of high-performance athletes, the detection of how similar responses are occurring in other countries and cultures will allow a general comparison of how athletes are coping with the global COVID-19 situation. While everyone hopes that this pandemic will not last long, similar situations in the future could mean the manifestation of behaviors and responses already experienced. Improved psychological resources to train and strengthen tolerance to dysfunctional responses (e.g., reframing challenges, self-regulating emotions, assuming other social roles) will generate a better management of athletes' desperate situations, preparing them not to maximize their discomfort about the development of their present and future sport.

## Data Availability Statement

The datasets presented in this article are not readily available because the data taken and recorded for this study are kept under the strictest care of the confidentiality of our institution and collaborating institutions. If you wish to review or use them, the authors must be expressly requested, under specific arguments, to obtain the relevant approvals. Requests to access the datasets should be directed to jgonzalez@ugr.es.

## Ethics Statement

The studies involving human participants were reviewed and approved by University of Granada. ID: 1494/2020. The patients/participants provided their written informed consent to participate in this study.

## Author Contributions

JG-H: introduction, methodology design, and analyses. MT-G, AY, and AN-L: translate, discussion, and conclusions. CL-M: methodology and analyses. All authors contributed to the article and approved the submitted version.

## Conflict of Interest

The authors declare that the research was conducted in the absence of any commercial or financial relationships that could be construed as a potential conflict of interest.

## References

[B1] Armstrong S. Oomen-Early J. (2009). Social connectedness, self-esteem, and depression symptomatology among collegiate athletes versus nonathletes. J. Am. Coll. Health 57, 521–526. 10.3200/JACH.57.5.521-526 19254893

[B2] Arnold R. Edwards T. Rees T. (2018). Organizational stressors, social support, and implications for subjective performance in high-level sport. Psychol. Sport Exerc. 39, 204–212. 10.1016/j.psychsport.2018.08.010

[B3] Bardeen J. R. Fergus T. A. (2016). Emotional distress intolerance, experiential avoidance, and anxiety sensitivity: the buffering effect of attentional control on associations with posttraumatic stress symptoms. J. Psychopathol. Behav. Assess. 38, 320–329. 10.1007/s10862-015-9522-x

[B4] Boffa J. W. Short N. A. Gibby B. A. Stentz L. A. Schmidt N. B. (2018). Distress tolerance as a mechanism of PTSD symptom change: evidence for mediation in a treatment-seeking sample. Psychiatr. Res. 267, 400–408. 10.1016/j.psychres.2018.03.085 29960937 PMC6434692

[B5] Brand R. Wolff W. Hoyer J. (2013). Psychological symptoms and chronic mood in representative samples of elite student-athletes, deselected student-athletes and comparison students. Sch. Ment. Health 5, 166–174. 10.1007/s12310-012-9095-8

[B6] Cece V. Guillet-Descas E. Nicaise V. Lienhart N. Martinent G. (2019). Longitudinal trajectories of emotions among young athletes involving in intense training centres: do emotional intelligence and emotional regulation matter? Psychol. Sport Exerc. 43, 128–136. 10.1016/j.psychsport.2019.01.011

[B7] Chalmers J. Quintana D. Abbott M. J. A. Kemp A. (2014). Anxiety disorders are associated with reduced heart rate variability: a meta-analysis. Front. Psychiatr. 5, 1–11. 10.3389/fpsyt.2014.00080 25071612 PMC4092363

[B8] Cheung T. Yip P. S. (2015). Depression, anxiety and symptoms of stress among Hong Kong nurses: a cross-sectional study. Int. J. Environ. Res. Public Health 12, 11072–11100. 10.3390/ijerph120911072 26371020 PMC4586662

[B9] Costa S. Santi G. di Fronso S. Montesano C. Di Gruttola F. Ciofi E. G.. (2020). Athletes and adversities: athletic identity and emotional regulation in time of COVID-19. Sport Sci. Health 16, 609–618. 10.1007/s11332-020-00677-9 32904823 PMC7457888

[B10] Davydov D. M. (2017). Alexithymia as a health risk and resilience factor. J. Psychosom. Res. 101, 66–67. 10.1016/j.jpsychores.2017.08.004 28867426

[B11] Daza P. Novy D. M. Stanley M. A. Averill P. (2002). The depression anxiety stress scale-21: Spanish translation and validation with a Hispanic sample. J. Psychopathol. Behav. Assess. 24, 195–205. 10.1023/A:1016014818163

[B12] Den Hartogh G. (2017). Suffering and dying well: on the proper aim of palliative care. Med. Health Care Philos. 20, 413–424. 10.1007/s11019-017-9764-3 28374105 PMC5569128

[B13] di Fronso S. Costa S. Montesano C. Di Gruttola F. Ciofi E. G. Morgilli L.. (2020). The effects of COVID-19 pandemic on perceived stress and psychobiosocial states in Italian athletes. Int. J. Sport Exerc. Psychol. Advance online publication. 10.1080/1612197X.2020.1802612

[B14] Donohue B. Gavrilova Y. Galante M. Gavrilova E. Loughran T. Scott J.. (2018). Controlled evaluation of an optimization approach to mental health and sport performance. J. Clin. Sport Psychol. 12, 234–267. 10.1123/jcsp.2017-0054

[B15] Drapeau A. Marchand A. Beaulieu-Prévost D. (2012). Epidemiology of psychological distress, in Mental Illnesses-Understanding, Prediction and Control, eds L'Abate L. (IntechOpen), 105–134. 10.5772/30872

[B16] Eccles D. W. Ward P. Woodman T. Janelle C. M. Le Scanff C. Ehrlinger J.. (2011). Where's the emotion? How sport psychology can inform research on emotion in human factors. Hum. Factors 53, 180–202. 10.1177/0018720811403731 21702335

[B17] Frank J. D. (1973). Persuasion and Healing: A Comparative Study of Psychotherapy. New York, NY: Johns Hopkins University Press.

[B18] Frank R. Nixdorf I. Beckmann J. (2015). Depression among elite athletes: prevalence and psychological factors. Dtsch Zeitschrift Sportmed. 64, 320–326. Available online at: https://www.germanjournalsportsmedicine.com/fileadmin/content/Englische_Artikel/Originalia_Frank_englisch.pdf20442052

[B19] Gorczynski P. F. Coyle M. Gibson K. (2017). Depressive symptoms in high-performance athletes and non-athletes: a comparative meta-analysis. Br. J. Sports Med. 51, 1348–1354. 10.1136/bjsports-2016-096455 28254747

[B20] Graupensperger S. Benson A. J. Kilmer J. R. Evans M. B. (2020). Social (Un) distancing: teammate interactions, athletic identity, and mental health of student-athletes during the COVID-19 pandemic. J. Adolesc. Health 67, 662–670. 10.1016/j.jadohealth.2020.08.001 32943294 PMC7489994

[B21] Grossbard J. R. Smith R. E. Smoll F. L. Cumming S. P. (2009). Competitive anxiety in young athletes: differentiating somatic anxiety, worry, and concentration disruption. Anxiety Stress Coping 22, 153–166. 10.1080/10615800802020643 18937102

[B22] Håkansson A. Jönsson C. Kenttä G. (2020). Psychological distress and problem gambling in elite athletes during covid-19 restrictions—a web survey in top leagues of three sports during the pandemic. Int. J. Environ. Res. Public Health 17:6693. 10.3390/ijerph17186693 32937978 PMC7559357

[B23] Haeberlein K. Evans L. Champaigne B. Handal P. J. (2020). Differences in distress and utilization of mental health services between 2005 and 2018: a potential trend? Psychiatr. Q. 91, 11–19. 10.1007/s11126-019-09692-7 31758300

[B24] Honkalampi K. De Berardis D. Vellante F. Viinamäki H. (2018). Relations between alexithymia and depressive and anxiety disorders and personality. in Alexithymia: Advances in Research, Theory, and Clinical Practice, eds Luminet O. Bagby R. M. Taylor G. J. (Cambridge: Cambridge University Press), 142–157. 10.1017/9781108241595.011

[B25] Junge A. Feddermann-Demont N. (2016). Prevalence of depression and anxiety in top-level male and female football players. BMJ Open Sport Exerc. Med. 2, 1–7. 10.1136/bmjsem-2015-000087 27900164 PMC5117057

[B26] Laborde S. Dosseville F. Allen M. S. (2016). Emotional intelligence in sport and exercise: a systematic review. Scand. J. Med. Sci. Sports 26, 862–874. 10.1111/sms.12510 26104015

[B27] Leary M. R. Tate E. B. Adams C. E. Batts Allen A. Hancock J. (2007). Self-compassion and reactions to unpleasant self-relevant events: the implications of treating oneself kindly. J. Personal. Soc. Psychol. 92:887. 10.1037/0022-3514.92.5.887 17484611

[B28] Madigan D. J. Hill A. P. Anstiss P. A. Mallinson-Howard S. H. Kumar S. (2018). Perfectionism and training distress in junior athletes: the mediating role of coping tendencies. Eur. J. Sport Sci. 18, 713–721. 10.1080/17461391.2018.1457082 29614917

[B29] Madigan D. J. Stoeber J. Passfield L. (2017). Perfectionism and training distress in junior athletes: a longitudinal investigation. J. Sports Sci. 35, 470–475. 10.1080/02640414.2016.1172726 27055481

[B30] Mannes Z. L. Waxenberg L. B. Cottler L. B. Perlstein W. M. Burrell L. E. II. Ferguson E. G.. (2019). Prevalence and correlates of psychological distress among retired elite athletes: a systematic review. Int. Rev. Sport Exerc. Psychol. 12, 265–294. 10.1080/1750984X.2018.1469162 31217807 PMC6583001

[B31] Neff K. D. Vonk R. (2009). Self-compassion versus global self-esteem: two different ways of relating to oneself. J. Personal. 77, 23–50. 10.1111/j.1467-6494.2008.00537.x 19076996

[B32] Nixdorf I. Frank R. Hautzinger M. Beckmann J. (2013). Prevalence of depressive symptoms and correlating variables among German elite athletes. J. Clin. Sport Psychol. 7, 313–326. 10.1123/jcsp.7.4.313

[B33] Páez D. Martínez-Sánchez F. Velasco C. Mayordomo S. Fernández I. Blanco A. (1999). Validez psicométrica de la Escala de Alexitimia de Toronto (TAS-20): un estudio transcultural. Bol. de Psicol. 63, 55–76.

[B34] Pensgaard A. M. Roberts G. C. (2000). The relationship between motivational climate, perceived ability and sources of distress among elite athletes. J. Sports Sci. 18, 191–200. 10.1080/026404100365090 10737270

[B35] Préville M. Potvin L. Boyer R. (1995). The structure of psychological distress. Psychol. Rep. 77, 275–293. 10.2466/pr0.1995.77.1.275 7501768

[B36] Putukian M. (2016). The psychological response to injury in student athletes: a narrative review with a focus on mental health. Br. J. Sports Med. 50, 145–148. 10.1136/bjsports-2015-095586 26719498

[B37] Reardon C. L. Hainline B. Aron C. M. Baron D. Baum A. L. Bindra A.. (2019). Mental health in elite athletes: International Olympic Committee consensus statement (2019). Br. J. Sports Med. 53, 667–699. 10.1136/bjsports-2019-100715 31097450

[B38] Rice S. M. Parker A. G. Mawren D. Clifton P. Harcourt P. Lloyd M.. (2020). Preliminary psychometric validation of a brief screening tool for athlete mental health among male elite athletes: the Athlete Psychological Strain Questionnaire. Int. J. Sport Exerc. Psychol. 18, 850–865. 10.1080/1612197X.2019.1611900

[B39] Rice S. M. Purcell R. De Silva S. Mawren D. McGorry P. D. Parker A. G. (2016). The mental health of elite athletes: a narrative systematic review. Sports Med. 46, 1333–1353. 10.1007/s40279-016-0492-2 26896951 PMC4996886

[B40] Roberts R. Woodman T. (2017). Personality and performance: moving beyond the Big 5. Curr. Opin. Psychol. 16, 104–108. 10.1016/j.copsyc.2017.03.033 28813330

[B41] Sandín B. Simons J. S. Valiente R. M. Simons R. M. Chorot P. (2017). Psychometric properties of the spanish version of The Distress Tolerance Scale and its relationship with personality and psychopathological symptoms. Psicothema 29, 421–428. 10.1037/t68244-000 28693717

[B42] Schinke R. Papaioannou A. Henriksen K. Si G. Zhang L. Haberl P. (2020). Sport psychology services to high performance athletes during COVID-19. Int. J. Sport Exerc. Psychol. 18, 269–272. 10.1080/1612197X.2020.1754616

[B43] Senişik S. Denerel N. Köyagasioglu O. Tunç S. (2020). The effect of isolation on athletes' mental health during the COVID-19 pandemic. Phys. Sportsmed. Advance online publication. 1–7. 10.1080/00913847.2020.1807297 33476217

[B44] Sherman N. (2014). Recovering lost goodness. Shame, guilt, and self-empathy. Psychoanal. Psychol. 31, 217–235. 10.1037/a0036435

[B45] Simons J. S. Gaher R. M. (2005). The distress tolerance scale: development and validation of a self-report measure. Motiv. Emotion 29, 83–102. 10.1007/s11031-005-7955-3

[B46] Steiner H. Denny K. Stemmle P. (2010). Adaptive styles in elite collegiate athletes: the role of activation and self-regulation. Personal. Ment. Health 4, 163–171. 10.1002/pmh.120

[B47] Sukys S. Tilindiene I. Cesnaitiene V. J. Kreivyte R. (2019). Does emotional intelligence predict athletes' motivation to participate in sports? Percept. Motor Skills 126, 305–322. 10.1177/0031512518825201 30665338

[B48] Turner M. J. Aspin G. Gillman J. (2019a). Maladaptive schemas as a potential mechanism through which irrational beliefs relate to psychological distress in athletes. Psychol. Sport Exerc. 44, 9–16. 10.1016/j.psychsport.2019.04.015

[B49] Turner M. J. Carrington S. Miller A. (2019b). Psychological distress across sport participation groups: the mediating effects of secondary irrational beliefs on the relationship between primary irrational beliefs and symptoms of anxiety, anger, and depression. J. Clin. Sport Psychol. 13, 17–40. 10.1123/jcsp.2017-0014

[B50] Vélez M. C. Palacio C. Moreno A. I. Krikorian A. (2013). Psychological and family-related facts of suffering in patients with chronic diseases. Techn. Reg. Anesth. Pain Manag. 17, 7–10. 10.1053/j.trap.2013.09.002

[B51] Walton C. C. Baranoff J. Gilbert P. Kirby J. (2020). Self-compassion, social rank, and psychological distress in athletes of varying competitive levels. Psychol. Sport Exerc. 50:101733. 10.1016/j.psychsport.2020.101733

[B52] Wolanin A. Hong E. Marks D. Panchoo K. Gross M. (2016). Prevalence of clinically elevated depressive symptoms in college athletes and differences by gender and sport. Br. J. Sports Med. 50, 167–171. 10.1136/bjsports-2015-095756 26782764

[B53] Woodman T. Cazenave N. Le Scanff C. (2008). Skydiving as emotion regulation: the rise and fall of anxiety is moderated by alexithymia. J. Sport Exerc. Psychol. 30, 424–433. 10.1123/jsep.30.3.424 18648114

[B54] Yousfi N. Bragazzi N. L. Briki W. Zmijewski P. Chamari K. (2020). The COVID-19 pandemic: how to maintain a healthy immune system during the lockdown–a multidisciplinary approach with special focus on athletes. Biol. Sport 37, 211–216. 10.5114/biolsport.2020.95125 32879542 PMC7433333

[B55] Zvolensky M. J. Vujanovic A. A. Bernstein A. Leyro T. (2010). Distress tolerance: theory, measurement, and relations to psychopathology. Curr. Dir. Psychol. Sci. 19, 406–410. 10.1177/0963721410388642 33746374 PMC7978414

